# Safety and efficacy of bleomycin/pingyangmycin-containing chemotherapy regimens for malignant germ cell tumor patients in the female genital system

**DOI:** 10.18632/oncotarget.15021

**Published:** 2017-02-02

**Authors:** Qianying Zhao, Dongyan Cao, Mei Yu, Jiaxin Yang, Yongjian Liu, Yang Xiang, Ming Wu, Lingya Pan, Jinghe Lang, Kaifeng Xu, Jiangna Han, Keng Shen

**Affiliations:** ^1^ Department of Gynecology and Obstetrics, Peking Union Medical College Hospital, Chinese Academy of Medical Sciences & Peking Union Medical College, Beijing, China; ^2^ Department of Respiratory Medicine, Peking Union Medical College Hospital, Chinese Academy of Medical Sciences & Peking Union Medical College, Beijing, China

**Keywords:** bleomycin, medication safety, treatment efficacy, pulmonary toxicity, malignant germ cell tumor

## Abstract

**Objectives:**

To comprehensively evaluate the safety and effectiveness of bleomycin/pingyangmycin-containing chemotherapy for female patients with malignant germ cell tumors in their genital system; to assess the diagnostic value of pulmonary function tests for bleomycin-induced pulmonary toxicity.

**Methods:**

Data from a cohort of 120 patients, collected across 25 years, was reviewed. Chemotherapy-related adverse events were routinely monitored. Pulmonary toxicity was diagnosed and graded according to serial pulmonary function testing results, and potential impact factors were explored. Short-term remission probability and long-term prognosis were evaluated.

**Results:**

Overall, 49.2% of the patients had pulmonary dysfunction, and the majority manifested as diffusion function impairment. A moderate reduction of carbon monoxide diffusion capacity was detected in 45.0% of all patients, and was severe in 3 patients. Thrombocytopenia, renal dysfunction, and accumulating dose of bleomycin/pingyangmycin significantly increased the risk of lung injury (*P*<0.05). Thorough surgical removal of tumors enhanced both remission and survival rate. Full-dose delivery of bleomycin/pingyangmycin and patients sensitivity to chemotherapy also improved long-term survival (*P*<0.05).

**Conclusions:**

BPT could be sensitively detected and elaborately graded by PFTs, but the appropriate cut-off value for diagnosis needs further investigations. Timely recognition and control of renal dysfunction and thrombocytopenia could avail the patients of the opportunity to complete curative antineopalstic treatment. Prescriptive bleomycin/pingyangmycin-containing chemotherapy after optimal surgical resection could benefit MGCT patients maximally by improving both remission and survival rate.

## INTRODUCTION

Malignant germ cell tumor (MGCT) is a rare entity in the female genital system and has a peak incidence in women under the age of 20 [1]. Due to cisplatin-based chemotherapy, the 5-year survival rate for patients with MGCTs exceeds 90% [2]. Given that 85% of metastatic GCTs are also cured and that the majority of patients are young adults, treatment-related complications become a core determinant for therapeutic plans [3].

Bleomycin is an attractive addition to combination chemotherapy regimens because of its broad activity and low myelotoxicity. The drug has been the mainstay for years in the treatment of MGCTs [4]. Meanwhile, bleomycin-induced pulmonary toxicity (BPT) has been commonly recognized since its clinical application from the early 1970s [5]. BPT may have immediate fatal effects or long-term clinical consequences for survivors with a long life expectancy [6].

However, consistent diagnostic criteria have yet to be developed. The incidence of BPT ranges from 2% to 46% due to diverse examination strategies that range from non-specific respiratory symptoms and less-sensitive radiographs to invasive lung biopsy. A number of potential risk factors for BPT have been revealed, including increasing age, underlying lung disease, smoking history, a cumulative bleomycin dose, renal insufficiency, radiation, supplemental oxygen exposure, and granulocyte colony-stimulating factor (G-CSF) support [3, 6, 7].

In addition, other therapeutic side effects (e.g., myelosuppression and renal dysfunction) might influence BPT severity as well as oncologists’ clinical decisions. Therefore, we reviewed the records from a continuous cohort of female MGCTs in the genital system and prospectively followed these patients. The aim of this study was to assess both the safety and efficacy of PEB (cisplatin, etoposide, and bleomycin/pingyangmycin) or PVB (cisplatin, vincristine, and bleomycin/pingyangmycin) chemotherapy with an emphasis on exploring the incidence and influential factors of BPT measured by serial pulmonary function test (PFT) results.

## PATIENTS AND METHODS

The Patients’ Record Database was searched for eligible patients between 1998 and 2014. Our final study group included all female patients with MGCTs in their genital system who were treated with a bleomycin/pingyangmycin-containing regimen at our institution. Pingyangmycin, isolated from actinomycetes in Pingyang County (China), has a similar structure to the composition A5 of bleomycin compound and presents equal antineoplastic effect. MGCT diagnoses were confirmed by the pathological review of all specimens in our hospital, and all histological subtypes were eligible for this study.

Patients with FIGO (International Federation of Gynecology and Obstetrics) stage-I dysgerminoma and immature teratoma (grade 1) could be followed, together with those who did not receive a bleomycin/pingyangmycin-containing regimen, were excluded. Standard front-line chemotherapy was either 3 or 4 cycles of PEB or PVB every 3 weeks (cisplatin 30-35 mg/m^2^/d on days 1-3, etoposide 100 mg/m^2^/d on days 1-3, or vincristine 1.0-1.5 mg/m^2^/d on days 1-2, bleomycin/pingyangmycin 15 mg/m^2^/d on days 1-2 or 20 mg/m^2^/d day 2/weekly). The actual cycles that were applied might be flexible based on disease condition and therapeutic response. Usually, two courses were consolidated after serum tumor makers (e.g., CA125, AFP and HCG) declined to a normal level. Bleomycin/pingyangmycin was discontinued if (1) the patient's carbon monoxide diffusion capacity corrected for hemoglobin (DLCO) dropped below 70% of the predicted value; or (2) decreased > 20% from baseline level during treatment; and if (3) the lifetime dose limit of 250 mg/m^2^ was exceeded. A regimen with bleomycin/pingyangmycin omitted (e.g. cisplatin and etoposide, with or without vincristine) was subsequently substituted if necessary. The initial treatment was considered to be effective if the neoplasm was optimally excised and if the surveillance indicators (tumor markers, imaging and physical examinations) were all negative for at least 4 weeks after the completion of treatment. Side effects were under surveillance: complete blood count, liver and renal function, chest X-ray, PFT, glomerular filtration rate (GFR) or creatinine clearance rate (CCr), and tumor markers were monitored. Patients who did not receive all their antineoplastic medications at our institution were omitted for inadequate records.

With PFTs we assessed the patients’ forced vital capacity (FVC), forced expiratory volume in 1 second (FEV1), FEV1/FVC ratio, total lung capacity (TLC,) and DLCO. According to the Global Initiative for Chronic Obstructive Lung Disease (GOLD) criteria [8], obstructive pulmonary function impairment was defined as an FEV1/FVC ratio < 0.70 and FEV1 < 80% of the predicted value. Restrictive pulmonary function impairment and diffusion capacity impairment were defined according to the Common Terminology Criteria for Adverse Events (CTCAE) version 3.0 [9, 10]. Specifically, restrictive dysfunction was defined as TLC < 75% of the predicted value. Diffusion impairment was measured using the DLCO and the disease severity was graded from 0 to 5: (0) no impairment, DLCO > 90% of the predicted value; (1) mild impairment, 90% ≥ DLCO > 75% of the predicted value; (2) moderate impairment, 75% ≥ DLCO > 50% of the predicted value; (3) severe and undesirable impairment, 50% ≥ DLCO > 25% of the predicted value; (4) life-threatening or disabling impairment, DLCO < 25% of predicted value; and (5) death. By definition, a pulmonary function impairment of grade 2 or higher ( ≤ 75%) can be considered abnormal and reflects physiological impairment [10]. Patients with underlying pulmonary dysfunction before chemotherapy were excluded.

Other treatment toxicities and potential risk factors for BPT were co-recorded, including age, prior lung disease, allergy history, renal function, cumulative bleomycin/pingyangmycin dose and delivery approach (intravenous, intramuscular or both), myelosuppression and the consequent use of G-CSF or blood transfusion and supplemental oxygen exposure. Tobacco and alcohol consumption information was not available for evaluation in our young female cohort. The lowest GFR or CCr was recorded and split by 80 ml/min for renal dysfunction diagnosis [3]. Aside from the total dose of bleomycin/pingyangmycin, the time when BPT initially occurred was also documented. Bone marrow toxicity was graded for each cycle according to CTCAE [9], and the worst degree of each myelosuppression subtype was applied for assessment. G-CSF and component blood transfusion (erythrocytes or thrombocytes) was prescribed accordingly. History of major surgery following chemotherapy was used as a surrogate identifier of supplemental oxygen use, which had been previously cited as a risk factor for BPT [3, 11].

The majority of patients admitted in the last decade are regularly followed-up at our Outpatient Department or local referral hospitals, and long-term prognosis was analyzed in this cohort. The overall survival (OS) and disease-free survival (DFS) were estimated with an emphasis on the association with BPT. Age was dichotomized by the median age of the study cohort, and the total dosage was categorized based on whether the course number of the bleomycin/pingyangmycin-containing regimen exceeded the guideline recommendations.

Influential factors for BPT severity were evaluated univariately by log-rank tests or Fisher's exact tests with 95% confidence interval (CI). Multivariate analysis was performed with the logistic regression method. Because of small event numbers, it was not possible to evaluate the effects of risk factors for obstructive and restrictive pulmonary function impairments. The observation time for OS ranged from the date of diagnosis to the date of death or the study end date, whichever occurred first. While the endpoint for DFS was either the date of first recurrence or the last follow-up starting from the completion of the first effective treatment. Potential clinical parameters for survival were analyzed univariately using the Kaplan-Meier method. A *P*-value < 0.05 (two-sided) was considered to be statistically significant. Statistical analyses were performed using Statistical Product and Service Solutions (SPSS) Statistics 20.0 (IBM Corporation, Armonk, New York, USA).

This study was approved by the institutional review board at Peking Union Medical College Hospital (PUMCH), Beijing, China, and the data of all patients were analyzed anonymously.

## RESULTS

### Study population

**Figure 1:**
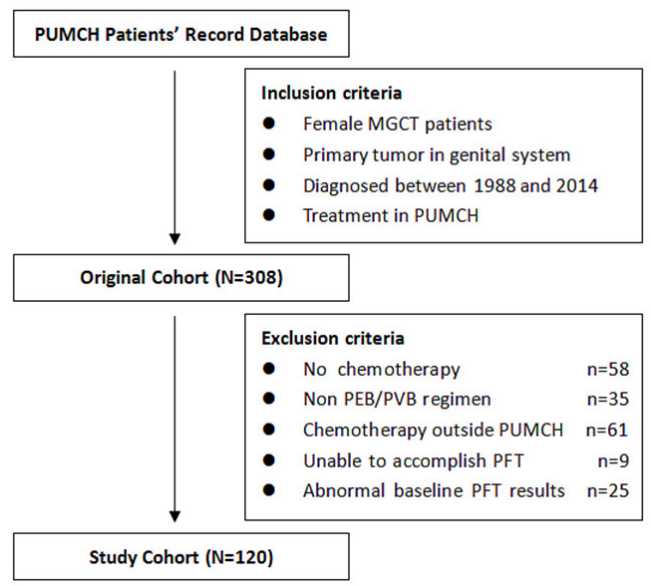
Process of patients’ enrollment for this study

A review of the database revealed 308 female patients with MGCTs in their genital system who were treated at PUMCH during the specified study period. Eventually, 120 cases met the criteria of our study, and the selection process and reasons for the exclusion of patients are summarized in Figure 1.

### Clinical characteristics

Table 1 shows the clinical features of the patients enrolled in the study, including four DSD (disorder of sex development) females (social gender). BPT incidence was similar in DSD and other patients (*P* = 1.000). The majority of the patients were Han Chinese who were diagnosed between age 10 and 30. Excluding 12 tumors with mix histologies, approximately 70% of the MGCTs were non-dysgerminomas: embryonal carcinoma (1), endodermal sinus tumor (32), gonadoblastoma (1), and immature teratoma (43), with dysgerminomas in the remaining 30%. Overall, 103 (85.8%) patients merely had a neoplasm confined within the pelvis (i.e., FIGO stage I/II). The majority (83.3%) of patients only received PEB regimen, and domestic pingyangmycin was chose for most patients (75.0%) because of economic reasons. Moreover, there were no significant differences found between the BPT and non-BPT group in terms of age, ethnic group, prior lung disease, allergic history, histology and stage (*P* > 0.05).

**Table 1:** Clinical characteristics of enrolled patients

Clinical features	All patients		BPT patients		Non-BPT patients		*P*-value
	No.	%	No.	%	No.	%	
Total	120	100.0	59	100.0	61	100.0	-
**Age, years**							
Median (Range)	20.7 (6-45)		21.2 (7-38)		20.3 (6-45)		0.984
**Ethnic group**							**1.000**
Han	111	92.5	55	93.2	56	91.8	
Minority	9	7.5	4	6.8	5	8.2	
**Prior lung disease**							**1.000**
Absent	119	99.2	59	100.0	60	98.4	
Present	1	0.8	0	0.0	1	1.6	
**Allergic history**							0.645
Absent	97	80.8	49	83.1	48	78.7	
Present	23	19.2	10	16.9	13	21.3	
**Histology**							0.302
Dysgerminoma	31	25.8	12	20.3	19	31.1	
Non-dysgerminoma	77	64.2	42	71.2	35	57.4	
Mixed MGCT	12	10.0	5	8.5	7	11.5	
**Stage**							1.000
FIGO I/II	103	85.8	51	86.4	52	85.2	
FIGO III/IV	17	14.2	8	13.6	9	14.8	
**Regimen**							0.051
PEB	100	83.3	48	81.3	52	85.3	
PVB	12	10.0	4	6.8	8	13.1	
Both	8	6.7	7	11.9	1	1.6	
**Agent**							0.555
Bleomycin	24	20.0	13	22.0	11	18.0	
Pingyangmycin	90	75.0	42	71.2	48	78.7	
Both	6	5.0	4	6.8	2	3.3	
**Route**							**0.026**
Intravenous	27	22.5	12	20.3	15	24.6	
Intramuscular	83	69.2	38	64.4	45	73.8	
Both	10	8.3	9	15.3	1	1.6	
**Schedule**							**0.018**
Weekly	60	50.0	24	40.7	36	59.0	
21-day	48	40.0	25	42.4	23	37.7	
Both	12	10.0	10	16.9	2	3.3	

Abbreviations: BPT, bleomycin induced pulmonary toxicity; FIGO, International Federation of Gynecology and Obstetrics; MGCT, malignant germ cell tumor; PEB/PVB, cisplatin, etoposide/ vincristine and bleomycin/ pingyangmycin

### Bleomycin/pingyangmycin induced pulmonary toxicity

**Figure 2:**
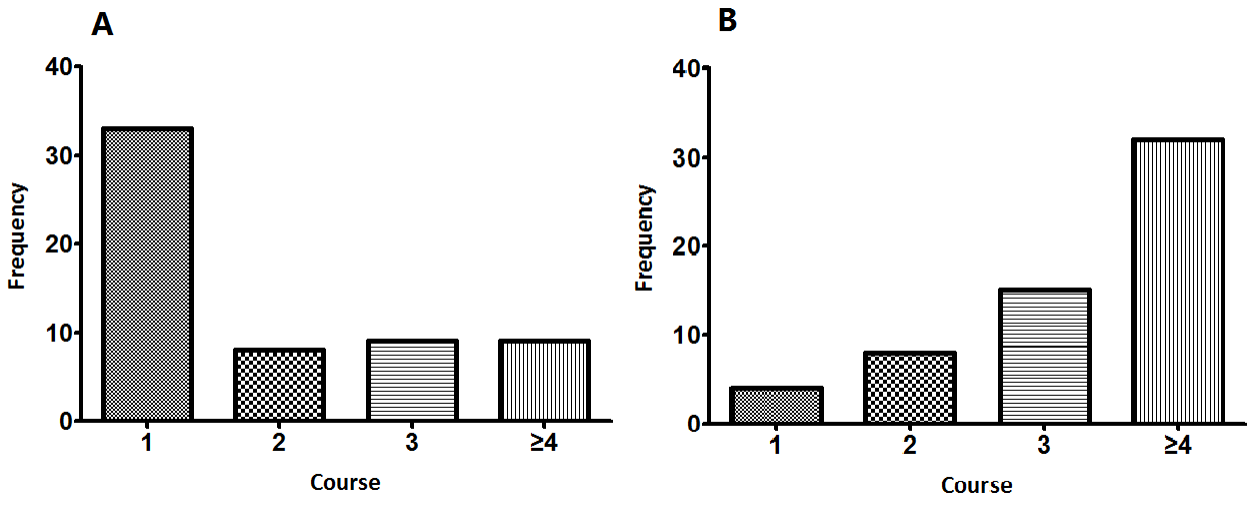
**The occurrence time of A. the initial BPT; B. the most severe BPT**.

Based on the worst PFT result of each patient from their first to the last administration of bleomycin/pingyangmycin, only one case had mild and transient obstructive dysfunction, which was accompanied by diffusion impairment. Another 2 patients merely had TLC that dropped below 75% of the predicted value with normal FEV1, FEV1/FVC and DLCO. Diffusion impairment was present in 57 patients, and the severity was moderate (grade 2) in 54 patients and severe (grade 3) in 3 patients. None had a diffusion capacity impairment of grade 4 or 5. In total, 59 patients (49.2%) out of the 120 in the cohort had BPT defined as obstructive or restrictive dysfunction, or decreased DLCO (grade 2 or higher). Only three BPT (grade 2) patients had symptoms (e.g., cough, expectoration or thoracalgia), whereas two from the non-BPT group also complained of similar discomfort. Among the BPT group, 21 patients had an abnormal chest X-ray or computed tomography (CT) images, including two patients with symptoms. However, the manifestation was diverse and non-specific, such as nodules, bands, effusion or bulla. A typical image of BPT as fibrosis and organization was found in only one patient through high resolution CT. This patient had grade 2 DLCO reduction and cough. Furthermore, three patients had hypersensitive BPT (grade 2-3), which was characterized by a prompt DLCO reduction with less than 60 mg/m^2^ of bleomycin/pingyangmycin administration. Usually, DLCO would immediately drop more than 15% from the baseline, and their pulmonary function would not recover, even after drug withdrawal. Taking no account of this unique reaction type, the average cumulative doses of bleomycin/pingyangmycin in the BPT and non-BPT group were significantly different (*P* < 0.05). The average accumulated dosages for BPT patients were 200.4 mg/m^2^ and 120 mg/m^2^ through the intramuscular and intravenous administration route, respectively, and both dosages were significantly higher than those of the non-BPT group. In addition, PFTs from 55.9% of BPT patients would present abnormality right after the first course, whereas 54.2% of these patients had their worst pulmonary function after four or more courses of PEB/PVB (Figure 2). The incidence of BPT was similar between groups applied distinct route of bleomycin/pingyangmycin administration (i.e. intravenous and intravascular), or either chemotherapeutic schedule (i.e. 3-week and weekly). However, patients who had interchanged regimens would be more likely to develop BPT (*P* < 0.05, Table 1).

### Other common adverse events from treatment

The lowest degrees of each bone marrow toxicity subtype for single patient were recorded and the pooled data were listed in Table 2. Myelosuppression in aspects of hemoglobin and thrombocyte was modest for the most part, whereas the worst granulopenia had reached a CTCAE grade 3 or 4 for 79.2% of patients. As a consequence, G-CSF was generally applied to patients with grade 3 or 4 granulopenia for therapeutic use, whereas prophylactic treatment was implemented for grade 4 cases. Patients with renal function impairment were 33.9% and 8.2% of the BPT and non-BPT groups, respectively. Abundant hydration was arranged due to cisplatin, and the urine volume should be no less than 2500 ml on the day cisplatin was administrated. Three patients suffered serious renal impairments (GFR < 60ml/min), and one of them had cisplatin dose induced, when the worst renal function was detected in the last chemotherapy cycle for the other two patients. Specifically, anemia and thrombocytopenia severity, together with renal insufficiency, significantly increased the risk of BPT by univariate evaluation (*P* < 0.05).

**Table 2:** Common chemotherapy-related adverse events

	All Patients		BPT Patients		Non-BPT Patients		*P*-value
	No.	%	No.	%	No.	%	
Total	120	100.0	59	100.0	61	100.0	-
**Bone Marrow toxicity (CTCAE grade)**							
**Hemoglobin**							**0.000**
≤1	77	64.2	33	55.9	44	72.1	
2	22	18.3	11	18.6	11	18.0	
3	13	10.8	8	13.6	5	8.3	
4	8	6.7	7	11.9	1	1.6	
**Leukocyte**							0.295
≤1	9	7.5	5	8.5	4	6.6	
2	16	13.3	10	16.9	6	9.8	
3	48	40.0	15	25.4	33	54.1	
4	47	39.2	29	49.2	18	29.5	
**Thrombocyte**							**0.020**
≤1	90	75.0	36	61.0	54	88.5	
2	14	11.7	10	16.9	4	6.6	
3	10	8.3	8	13.6	2	3.3	
4	6	5.0	5	8.5	1	1.6	
**Renal dysfunction (GFR or CCr)**							**0.001**
>80	95	79.2	39	66.1	56	91.8	
≤80	25	20.8	20	33.9	5	8.2	

Abbreviations: BPT, bleomycin/pingyangmycin induced pulmonary toxicity; CTCAE, common terminology criteria for adverse events; GFR, glomerular filtration rate; CCr, creatinine clearance rate

### Impact factors for BPT severity

BPT severity was graded based on the degree of DLCO decline. Risk factors were explored in regards to epidemiologic information, individual history, chemotherapy details, concomitant side effects and combined treatments. Table 3 depicts the results of univariate analyses for each potential hazard, excluding three hypersensitive BPT cases. The cumulative dose of bleomycin/pingyangmycin, the patients’ renal function, hemoglobin and thrombocyte value, platelet transfusion, and chemotherapy regimen (PEB or PVB, weekly or monthly), and the administration route of bleomycin/pingyangmycin were significantly associated with the degree of BPT (*p* < 0.05). However, multivariate analysis revealed that only increasing dosage, renal insufficiency and serious thrombocytopenia independently aggravated BPT after adjusting for other potential impact factors (*P* < 0.05).

**Table 3:** Univariate analysis of influential factors for BPT severity

Impact factors	*P*-value	Impact factors	*P*-value
Epidemiologic information		Chemotherapy detail	
Age at diagnosis	0.335	PEB/PVB	**0.043**
Ethnic group	0.856	Bleomycin/pingyangmycin	0.755
**Individual history**		Weekly/monthly	**0.031**
Prior lung disease	1.000	Route of administration	**0.041**
Allergic history	0.603	Cumulative dose	**0.000**
**Disease state**		Average course interval	0.256
Stage of disease	0.081	**Combined treatment**	
Histological subtype	0.741	G-CSF	0.331
**Side effects of treatment**		Antibiotics	0.904
Renal dysfunction	**0.001**	Blood transfusion	-
Drug allergy	0.332	Erythrocyte	0.116
Granulopenia	0.176	Thrombocyte	0.035
Anemia	**0.041**	Chalybeate	0.067
Thrombocytopenia	**0.000**	Post-chemotherapy surgery	0.072

Abbreviations: BPT, bleomycin-induced pulmonary toxicity; PEB/PVB, cisplatin, etoposide/ vincristine and bleomycin; G-CSF, granulocyte colony stimulating factor

Eighty percent of the patients with impaired renal eliminating ability had pathological DLCO reduction, whereas the proportion was merely 41.1% among patients with normal renal function. Moreover, all the three patients with severe BPT (grade 3) had a platelet level dropped below 75*10^9^/L. Specifically, patients who had a grade 2 or higher degree of thrombocytopenia had an odds ratio (OR) of 4.93 for BPT occurrence compared to the combined normal and grade 1 group (*P* < 0.05).

### Remission probability and long-term prognosis

With a satisfactory surgical resection of solid tumors and standard frontline chemotherapy, most patients (94.2%) achieved remission immediately. However, the disease persisted for 7 remaining patients (5.8%). Potential impact factors for remission probability detected univariately are listed in Table 4. However, multivariate analysis demonstrated that only surgery thoroughness was an independent predictor for short-term response (*P* < 0.05). Specifically, the remission probability for patients who had an optimal cytoreductive surgery was 9.9 times higher compared to patients whose tumors were not completely removed. In addition, BPT altered the oncologists’ clinical decision: bleomycin/pingyangmycin dosage had been reduced in 42.3% among the BPT group (20-60 mg/m^2^ intramuscularly and 10-30 mg/m^2^ intravenously), whereas the remaining BPT patients had completed full-dose administration. More importantly, the latter group was more likely to remit with a curative remedy (OR 1.190, 95% CI 1.003-1.413) without the exacerbation of BPT.

**Table 4:** Impact factors for remission and prognosis

Impact factors	*P*-value		
	Remission probability	Overall survival	Disease-free survival
Age at diagnosis	0.544	0.827	0.287
Ethnic group	0.429	0.610	0.211
Stage	0.258	0.247	0.384
Histological subtype	0.721	0.318	0.180
Thoroughness of surgery	**0.039**	**0.009**	0.711
PEB/PVB	0.487	0.956	0.093
Bleomycin/pingyangmycin	**0.043**	0.420	0.197
Route of administration	0.649	0.744	0.645
Weekly/monthly	0.334	0.238	0.660
Course interval	0.369	0.911	0.242
Cumulative dose	0.069	**0.003**	0.393
Renal dysfunction	**0.034**	0.294	0.384
BPT severity	0.370	0.214	0.806
Granulopenia	0.343	0.610	0.464
Anemia	**0.003**	0.130	0.127
Thrombocytopenia	1.000	0.378	0.339
Remission	-	**0.000**	-

Abbreviations: BPT, bleomycin-induced pulmonary toxicity; PEB/PVB, cisplatin, etoposide/ vincristine and bleomycin

After a median observation time of 53.2 months (range, 2.1 to 139.7 months), two patients died due to ovarian cancer. The 5-year OS rate was predicted to be 97.3% with an estimated median OS period of 11.2 years. Table 4 depicts the results of prognostic factors for OS investigated univariately. It indicated that optimal cytoreduction, sufficient delivery of bleomycin/pingyangmycin and the initial response to therapy significantly improved survival (*P* < 0.05). Specifically, both deceased patients failed to achieve remission after the initial treatment. Until the end of the study, three patients had recurring diseases with a median relapse interval of 2.7 years. However, no prognostic factors that influenced recurrence were discovered, which probably occurred due to current limited events.

## DISCUSSION

Diagnosed by PFTs, approximately half of the patients have developed pathological pulmonary dysfunction at our institution. In particular, diffusion capacity impairment was prevalent, whereas obstructive and restrictive function impairments were observed in only a few cases. BPT has been recognized ever since its early clinical trials, the diagnostic criteria have not yet been unified. Due to the diversity of examination strategies and enrolled populations, the incidence in prior studies ranged from 2% to 46% in various studies [12]. Compared to respiratory symptoms and chest radiographs, PFT showed higher sensitivity. However, some researchers blamed PFT for vast false positive results [13]. Though life-threatening BPT and cause-specific deaths were prevented from happening, the remission probability was decreased for our BPT patients who had a reduced-dose of bleomycin/pingyangmycin administration. We thus posited that grade 2 DLCO reduction ( > 25%) would better be merely a warning sign, rather than an indicator for immediate drug cessation. On the other hand, a more precise grading of BPT severity might help determine a more appropriate cut-off value for clinical intervention. Specifically, consultation with specialists in respiratory medicine is valuable.

Other common chemotherapy-related complications were routinely monitored to maintain a safe level of medication. The most frequent and prominent myelosuppression was granulopenia, and G-CSF was prescribed when WBC dropped too much or too soon, or without timely recovery. Severe anemia and thrombocytopenia also deserve attention, and component blood transfusion should be supplemented accordingly. Renal function was seriously impaired in three patients, probably due to cisplatin. Urine volume is supposed to be monitored, and adequate hydration should be applied. Carboplatin may be a substitute when cisplatin had to be ceased.

In this study, we focused on the relationship between BPT and other side effects, which has seldom been assessed in other studies. To the best of our knowledge, hand-in-hand aggravation of thrombocytopenia and BPT was revealed for the first time. However, the mechanism underlying this interaction is unknown. Lung fibrosis and inflammatory reaction might lead to platelet consumption, or disordered coagulation and fibrinolysis may be important for the pathogenesis of pulmonary fibrosis [14, 15]. We suggest timely management of severe thrombocytopenia, which might bring both circulatory and respiratory adverse events under better control. However, the definite clinical value of this relationship needs further investigation and validation. Between 50% and 70% of bleomycin is excreted unchanged by the kidneys. The half-life is 2-5 h in patients with normal renal function, which can increase to 30 hours in patients with reduced GFRs [16], and lead to longer lung exposure to the drug. In accordance with other studies [3, 12], our results demonstrated that decreased GFR or CCr was a predictor for BPT. Renal function can be compromised in a number of ways, especially by cisplatin, or by a secondary obstruction due to the abdominal tumor. In addition, our study confirmed there were dose-dependent effects for BPT severity detected by other studies [3, 12, 17].

We also investigated other potential impact factors for BPT [2, 3, 6, 7, 12, 17–19], yet none of these factors were validated by our data. In particular, Gerson et al demonstrated that patients who received bleomycin in a continuous infusion did not have DLCO alterations, and other studies showed that continuous infusion might have less toxicity than bolus administration [12, 19]. We failed to find a significant difference in BPT severity between single intravenous and intramuscular routes. However, lung injury might be worse when patients received both administration methods successively. Therefore, we suggest a consistent single delivery pattern of bleomycin/pingyangmycin for each patient.

After all, the therapeutic efficacy is the primary concern, especially for a malignant disease with a good prognosis. Under standard guidelines, both the short-term remission and long-term survival rates were relatively high in our patients. Martin et al demonstrated that BPT resulted in a significant decrease in 5-year OS in patients with Hodgkin's lymphoma [6]. However, none of the treatment complications could explain the deaths in our study, which agreed with the results from Thomsen et al [2]. It is worth mentioning that the reduction of bleomycin/pingyangmycin administration did have a reversed impact on remission probability in the BPT group, whereas the dosages within lifetime limit generally would not lead to lethal BPT. Moreover, sufficient delivery of bleomycin/pingyangmycin improved OS. Collectively, a full-dose standard PEB/PVB regimen is recommended, and a more practicable BPT diagnostic model should be established using prospective controlled diagnostic trials.

We acknowledge that our study has several limitations. For the sake of accuracy, a large number of transferred patients without systematic records of side reactions were excluded. The cohort effects might influence the estimates, and factors underlying non-prescriptive chemotherapy might affect the drug-induced toxicities. Secondly, even though BPT was monitored while bleomycin/pingyangmycin was given, the impact factors were simultaneously collected. Therefore, the causal relationship could hardly be confirmed in a chronological manner. In addition, long-term complications need more intensive observation.

In conclusion, BPT could be sensitively detected and elaborately graded by PFTs, but the testing specificity needs to be enhanced. Concomitant thrombocytopenia, renal dysfunction, and an excessive dose of bleomycin/pingyangmycin significantly aggravate BPT. Therefore, simple and minimally invasive tests, such as a routine blood test, GFR and PFT should be monitored. With timely recognition and definitive control of treatment complications, patients would have more opportunities to be cured by integral PEB/PVB chemotherapy. Eventually, precision medicine could be achieved from a clinical perspective with treatment efficacy and safety comprehensively balanced for each patient.
